# Risk factor analysis and prediction model construction for severe adenovirus pneumonia in children

**DOI:** 10.1186/s13052-024-01771-1

**Published:** 2024-10-09

**Authors:** Yaowen Liang, Jinhuan Wu, Gang Chen, Yuchen Du, Yi Yan, Shuqin Xie, Wenxian Qian, Apeng Chen, Changhua Yi, Man Tian

**Affiliations:** 1 The Second Hospital of Nanjing, Affiliated Hospital to Nanjing University of Chinese Medicine, Nanjing, China; 2 Department of Intensive Care Unit, Children’s Hospital of Nanjing Medical University, Nanjing, China; 3 Children’s Hospital of Nanjing Medical University; The Second Hospital of Nanjing, Affiliated Hospital to Nanjing University of Chinese Medicine, Nanjing, China; 4 Department of Respiratory, Children’s Hospital of Nanjing Medical University, Nanjing, China

**Keywords:** Human adenovirus, Pneumonia, Children, Model

## Abstract

**Background:**

Severe adenovirus pneumonia in children has a high mortality rate, but research on risk prediction models is lacking. Such models are essential as they allow individualized predictions and assess whether children will likely progress to severe disease.

**Methods:**

A retrospective analysis was performed on children with adenovirus pneumonia who were hospitalized at the Children’s Hospital of Nanjing Medical University from January 2017 to March 2024. The patients were grouped according to clinical factors, and the groups were compared using Ridge regression and multiple logistic regression to identify risk factors associated with severe adenovirus pneumonia. A prediction model was constructed, and its value in clinical application was evaluated.

**Results:**

699 patients were included in the study, with 284 in the severe group and 415 in the general group. Through the screening of 44 variables, the final risk factors for severe adenovirus pneumonia in children as the levels of neutrophils (OR = 1.086, 95% CI: 1.054‒1.119, *P* < 0.001), D-dimer (OR = 1.005, 95% CI: 1.003‒1.007, *P* < 0.001), fibrinogen degradation products (OR = 1.341, 95% CI: 1.034‒1.738, *P* = 0.027), B cells (OR = 1.076, 95%CI: 1.046‒1.107, *P* < 0.001), and lactate dehydrogenase (OR = 1.008, 95% CI: 1.005‒1.011, *P* < 0.001). The value of the area under the receiver operating characteristic curve was 0.974, the 95% CI was 0.963–0.985, and the P-value of the Hosmer-Lemeshow test was 0.547 (*P* > 0.05), indicating that the model had strong predictive power.

**Conclusion:**

In this study, the clinical variables of children with adenovirus pneumonia were retrospectively analyzed to identify risk factors for severe disease. A prediction model for severe disease was constructed and evaluated, showing good application value.

## Introduction

Human adenovirus (HAdV) is the causative agent of adenovirus pneumonia (AVP), a common respiratory disease frequently reported among children under five [1, 2]. While AVP can occur at any time, the incidence can vary due to regional differences in climatic conditions and virus subtypes. In various countries and regions, adenovirus epidemics are often accompanied by co-infection [3]. Recent adenovirus epidemics have been more serious in China, with local outbreaks in winter and summer [4]. Among the 116 serotypes in groups A-G, HAdV-B7 and HAdV-B3 have been found to be the predominant subtypes causing infection in children [5].

While no significant change has been documented in the evolutionary characteristics and severity of adenovirus infections among children, reports have described its epidemic outbreak [6, 7]. Given the incomplete development in the immune system of children and the lack of specific antiviral agents, AVP rapidly progresses after infection and, in some cases, can even exacerbate severe adenovirus pneumonia (SAP). Despite continuous improvements in medical standards and treatment options for SAP, the prognosis of children with SAP remains poor due to the severity and rapid progression of the disease. SAP affects multiple systems and has a high mortality rate, exceeding 50% in cases of untreated SAP [8, 9]. Complications of SAP in children are numerous and may involve multiple organs, thereby leading to severe respiratory failure, toxic encephalopathy, and even acute respiratory distress syndrome (ARDS) and multiple organ dysfunction syndrome (MODS) [10]. SAP not only adversely affects the psychological well-being of the affected children, but also places a serious burden on families and society. Therefore, the early identification of SAP and timely intervention can reduce the risk of poor prognosis and even death in children. Herein, we comprehensively analyzed the data collected from children enrolled in the study and constructed a prediction model for SAP by comparing the clinical characteristics of mild and severe AVP infections. To promptly identify cases that may progress to SAP, timely targeted treatment was given to improve the prognosis of children.

## Materials and methods

### Study population

**Fig. 1:**
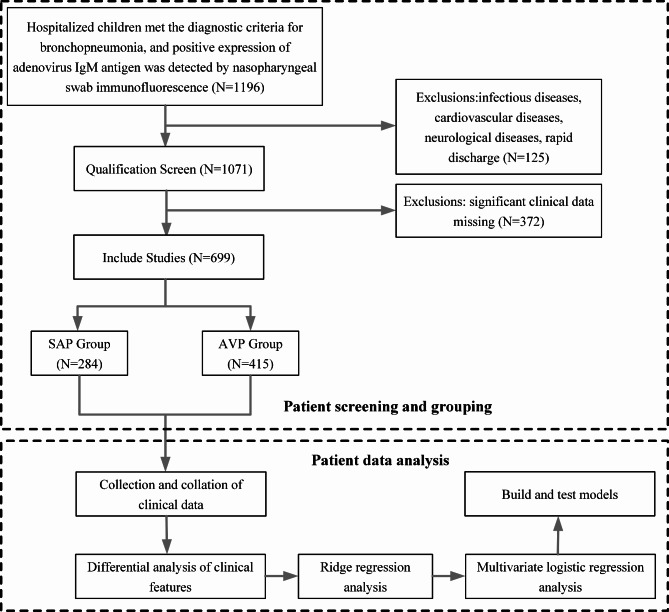
Flowchart of the study. The upper part shows the screening and grouping of patients, and the lower part shows the analysis of patient data. The two sections are in separate dotted boxes

This retrospective observational study was conducted on 699 children with APV who received treatment at the Nanjing Medical University Affiliated Children’s Hospital between January 2017 and March 2024. The patients were included in the study if they met the following criteria: age below 14 years; fulfill the diagnostic criteria for bronchopneumonia; positive expression of adenovirus immunoglobulin M (IgM) antigen, as tested by nasopharyngeal swab immunofluorescence method; and availability of complete clinical data. Patients were excluded if they met the following exclusion criteria: incomplete clinical data; cured within 24 h; infectious encephalitis or other infectious diseases; congenital heart disease (CHD) or other cardiovascular system diseases; inexplicable convulsions, epilepsy, or other neurological disorders; cancer (Fig. 1).

### Ethics statement

This study was conducted following the Declaration of Helsinki and has been reviewed and approved by the Medical Ethics Committee of the Children’s Hospital of Nanjing Medical University (202406011-1), and informed consent is exempted. We need to state that there is no risk to the child in this study, and the data on the course of the disease and the child’s examination are obtained through the hospital’s electronic medical record system.

### Diagnostic criteria

#### Diagnostic criteria for bronchopneumonia

The patient presents with respiratory symptoms, such as the recent onset of cough, sputum production, or worsening of pre-existing respiratory symptoms, with purulent sputum; fever and increased respiratory rate; auscultation of the lungs shows fixed moist rales and chest imaging suggests pneumonia; etiological analysis can determine the type of infection.

#### Diagnostic criteria for severe pneumonia

In terms of China’s national conditions and the guidelines for community-acquired pneumonia in children issued by the Chinese Society of Respiratory Diseases, children with pneumonia are diagnosed and classified into severe group if they have one or more of the following conditions at the time of admission: 1 Impaired consciousness; 2 Poor general physical condition; 3 Dyspnea, cyanosis, or tachypnea, with marked increases in the respiratory rate (infants > 70 bpm, children over 1 year old > 50 bpm); 4 Oxygen saturation < 92%, PaO_2_ < 60 mmHg, PaO_2_/FiO_2_ < 300 or requiring mechanical ventilation to assist breathing; 5 Persistent moderate-high fever for 10 days (temperature 38‒41℃) or ultra-high fever (temperature > 41 °C); 6 Chest imaging showing damage to multiple lobes, pleural effusion, pulmonary necrosis, or an increase in lung lesions of more than 50% within 48 h of admission; 7 Occurrence of complications such as septic shock and acute renal failure; 8 Absolute indication for transfer to ICU for treatment.

### Study design

#### Clinical data collection

According to the disease diagnostic criteria, the enrolled children were categorized into severe (SAP) and common (AVP) groups. The variables collected were data on basic information (including first admission data, admission physical examination, and discharge diagnosis), co-infection, and laboratory tests. Essential information included gender, age, weight, pulse, thermal spike (the highest value of the child’s axillary body temperature during the disease), underlying diseases, and complications (diagnosed by doctors as respiratory failure, heart failure, sepsis). Data on co-infection included nucleic acid testing for pathogens (*Mycoplasma pneumoniae*, influenza virus, respiratory syncytial virus) and bacterial cultures. Laboratory tests comprised blood routine (C-reactive protein, White blood cell, Lymphocyte, Neutrophil, Lymphocyte count, Monocytes count, Red blood cell and Hemoglobin concentration), coagulation (Prothrombin time, International normalized ratio, Activated partial Thromboplastin time, Fibrinogen, Thrombin time, D-dimer and Fibrinogen degradation products), biochemical (Alanine aminotransferase, Aspartate aminotransferase, Lactate dehydrogenase, Creatine kinase, Creatine kinase- MB, Total protein, Albumin, Globulin, Albumin-to-Globulin ratio, Glucose, Total bilirubin, Indirect bilirubin and Direct bilirubin), and lymphocyte percentage tests (T lymphocyte, B lymphocyte and Natural killer cell). In the results of this study, the names of statistical variables are presented in abbreviations. (Table 1)

**Table 1:** Full names and abbreviations for some statistical variables

Variables	Variables Abbreviations	Variables	Variables Abbreviations
Total protein	TP	Hemoglobin concentration	HGB
T lymphocyte	T cell	Prothrombin time	PT
Fibrinogen	FIB	International normalized ratio	INR
C-reactive protein	CRP	Activated partial Thromboplastin time	APTT
White blood cell	WBC	Thrombin time	TT
lymphocyte	LY	Fibrinogen degradation products	FDP
Neutrophil	NEUT	Alanine aminotransferase	ALT
Lymphocyte count	LYOE	Aspartate aminotransferase	AST
Monocytes count	MONOOE	Lactate dehydrogenase	LDH
Red blood cell	RBC	Albumin- to- Globulin ratio	AG
Creatine kinase	CK	Glucose	GLU
Creatine kinase- MB	CKMB	Total bilirubin	TBIL
Albumin	ALB	Direct bilirubin	DBIL
Globulin	GLB	B lymphocyte	B cell
Indirect bilirubin	IBIL	Length of stay	LOS
Natural killer cell	NK	Influenza	FLU
Mycoplasma	MP	Respiratory syncytial virus	RSV
Epstein-Barr virus	EB		

#### Statistical methods

Data arrangement and logistic analysis were completed with SPSS 27.0, Ridge regression applied R version 4.3.0 analysis, and model establishing was based on Storm Statistical Platform (www.medsta.cn/software) and R version 4.3.0 (2023-04-21). Counting data of the research was represented by the number of cases and percentage, adopting the χ2 test in inter-group comparison. Counting data that conformed to normal distribution was represented by mean ± standard error (x ± ns), adopting T-test. Counting data that conformed to non-normal distribution was represented by [M (P25, P75)], adopting the Mann-Whitney test. *P* ≤ 0.05 meant the difference had statistical significance.

To obtain the risk factors of SAP in children, we re-analyzed the differentiation using the Ridge regression method with the variables of *P* < 0.05. The data were imported and processed using the Foreign and Tidyr packages of the R version, and the Ridge regression chart was plotted with the Glmnet package, then executed multivariate logistic analysis to the variation that Coefficient > 0 in Ridge regression, and finally determined predictive factors of severe illness of SAP. Eventually, we constructed a nomogram and pictured the curve of receiver operating characteristic (ROC), evaluating the calibration degree and discrimination of the model with goodness of fit index (Hosmer- Lemeshow test) and area under the curve (AUC), in which *P*>0.05 (Hosmer- Lemeshow test ) indicated calibration degree of the model was reasonably good. (Fig. 1)

## Results

### Basic data

The study was conducted on 699 children, including 284 cases (40.63%) of SAP and 415 cases (59.37%) of AVP. The two groups showed no significant differences in age, gender, and disease characteristics (*P* > 0.05). The pulse elevated while hospitalized, and the average number of days of hospitalization was seven. The values of these two parameters were significantly higher in the SAP group than in the AVP group (*P* < 0.05). Overall, 189 patients from the SAP group had mixed infection with Mp (33.10%). Further, 74 (26.06%) patients had mixed infections with FLU and RSV, 32 (11.27%) cases had complicated respiratory failure and heart failure, and 17 (5.99%) cases had complicated sepsis.

### Laboratory analysis

In routine blood examination, CRP, WBC, and NEUT levels in the SAP group were significantly higher than those reported in the AVP group, while LY and RBC levels in the SAP group were lower than those in the AVP group. In the coagulation function test, the levels of D-dimer and FDP were significantly higher, and those of INR and APTT were lower in the SAP group than in the AVP group. In the biochemical function examination, the SAP group had significantly higher ALT, AST, LDH, CK, CKMB, GLB, and GLU levels but lower ALB and AG levels than the AVP group. The lymphocyte ratio test result revealed a significantly higher proportion of B cells and a lower proportion of T and NK cells in the SAP group. The differences in the above variables were statistically significant (*P* < 0.05) (Table 2).

**Table 2:** Comparative analysis of clinical data and laboratory test data of children with adenovirus pneumonia in the two groups

Variables Abbreviations (unit)	Total (*n* = 699)	AVP (*n* = 415)	SAP (*n* = 284)	Statistic	*P*
Pulse, Mean ± SD	127.66 ± 20.52	123.67 ± 18.59	133.50 ± 21.81	t=-6.21	< 0.001
TP (g/L), Mean ± SD	67.20 ± 6.60	67.47 ± 6.29	66.80 ± 7.03	t = 1.29	0.199
T cell (%), Mean ± SD	61.02 ± 13.06	64.32 ± 11.09	56.19 ± 14.19	t = 8.10	< 0.001
Age (Months), M (Q₁, Q₃)	35.00 (17.00, 59.00)	35.00 (18.00, 56.00)	35.50 (16.00, 64.00)	Z=-0.02	0.982
CRP (mg/L), M (Q₁, Q₃)	8.00 (7.88, 19.07)	8.00 (6.10, 14.43)	11.46 (8.00, 27.23)	Z=-5.81	< 0.001
WBC (10^9/L), M (Q₁, Q₃)	10.82 (7.83, 15.21)	9.36 (7.36, 12.78)	13.18 (9.88, 17.65)	Z=-8.88	< 0.001
LY (%), M (Q₁, Q₃)	35.00 (21.35, 50.55)	43.50 (28.05, 56.25)	25.25 (15.80, 37.02)	Z=-10.78	< 0.001
MONO (%), M (Q₁, Q₃)	8.00 (5.90, 10.50)	8.50 (6.35, 11.00)	7.40 (5.38, 9.62)	Z=-4.66	< 0.001
NEUT (%), M (Q₁, Q₃)	55.20 (37.60, 69.15)	45.80 (32.55, 61.00)	64.00 (53.32, 75.30)	Z=-11.12	< 0.001
LYOE (10^9/L), M (Q₁, Q₃)	3.28 (2.23, 5.04)	3.78 (2.58, 5.36)	2.64 (1.70, 4.32)	Z=-6.63	< 0.001
MONOOE (10^9/L), M (Q₁, Q₃)	0.83 (0.53, 1.25)	0.82 (0.54, 1.23)	0.83 (0.50, 1.25)	Z=-0.05	0.963
RBC (10^12/L), M (Q₁, Q₃)	4.47 (4.21, 4.69)	4.48 (4.25, 4.71)	4.43 (4.14, 4.66)	Z=-2.52	0.012
HGB (g/L), M (Q₁, Q₃)	121.00 (113.50, 127.00)	121.00 (114.00, 127.00)	120.00 (112.00, 127.00)	Z=-1.16	0.245
PT (s), M (Q₁, Q₃)	11.70 (11.00, 12.70)	11.70 (11.10, 12.80)	11.80 (11.00, 12.62)	Z=-1.04	0.297
INR (ratio), M (Q₁, Q₃)	1.04 (0.98, 1.13)	1.05 (0.99, 1.14)	1.04 (0.97, 1.12)	Z=-2.35	0.019
APTT (s), M (Q₁, Q₃)	32.40 (29.20, 36.45)	32.60 (29.94, 36.50)	31.80 (28.58, 36.30)	Z=-2.15	0.032
FIB (g/L), M (Q₁, Q₃)	3.23 (2.68, 3.75)	3.23 (2.73, 3.75)	3.19 (2.65, 3.76)	Z=-0.71	0.480
TT (s), M (Q₁, Q₃)	14.90 (13.80, 16.30)	14.90 (13.90, 16.10)	15.05 (13.70, 16.60)	Z=-0.84	0.401
D-dimer (ng/mL), M (Q₁, Q₃)	300.00 (198.00, 665.00)	222.00 (162.50, 288.00)	801.50 (468.00, 1455.25)	Z=-18.71	< 0.001
FDP (ug/mL), M (Q₁, Q₃)	2.52 (1.25, 4.67)	1.64 (0.99, 2.67)	5.45 (3.11, 9.45)	Z=-16.60	< 0.001
ALT (U/L), M (Q₁, Q₃)	16.00 (12.00, 25.00)	14.00 (11.00, 20.00)	20.50 (13.75, 34.25)	Z=-7.54	< 0.001
AST (U/L), M (Q₁, Q₃)	32.00 (25.00, 45.00)	29.00 (24.00, 37.00)	41.00 (29.00, 63.00)	Z=-8.63	< 0.001
LDH (U/L), M (Q₁, Q₃)	336.00 (270.00, 518.50)	291.00 (252.00, 334.50)	560.00 (426.00, 734.50)	Z=-17.56	< 0.001
CK (U/L), M (Q₁, Q₃)	62.00 (42.00, 91.50)	61.00 (42.00, 82.00)	62.00 (41.00, 113.00)	Z=-2.15	0.032
CKMB (U/L), M (Q₁, Q₃)	26.00 (21.00, 34.00)	23.00 (19.00, 29.00)	31.50 (26.00, 42.00)	Z=-10.65	< 0.001
ALB (g/L), M (Q₁, Q₃)	40.60 (37.70, 43.30)	41.90 (39.60, 44.40)	38.40 (34.50, 41.23)	Z=-11.11	< 0.001
GLB (g/L), M (Q₁, Q₃)	26.50 (23.30, 30.40)	25.50 (22.70, 28.90)	28.00 (24.40, 32.52)	Z=-6.37	< 0.001
AG (ratio), M (Q₁, Q₃)	1.51 (1.29, 1.79)	1.65 (1.43, 1.90)	1.33 (1.13, 1.57)	Z=-10.24	< 0.001
GLU (mmol/L), M (Q₁, Q₃)	4.91 (4.36, 5.68)	4.80 (4.30, 5.46)	5.09 (4.43, 6.06)	Z=-3.52	< 0.001
TBIL (umol/L), M (Q₁, Q₃)	4.50 (3.30, 6.13)	4.50 (3.30, 6.05)	4.50 (3.40, 6.23)	Z=-0.74	0.458
DBIL (umol/L), M (Q₁, Q₃)	2.01 (1.56, 2.71)	2.00 (1.50, 2.60)	2.10 (1.60, 2.90)	Z=-1.51	0.130
IBIL (umol/L), M (Q₁, Q₃)	2.60 (1.61, 3.60)	2.60 (1.70, 3.58)	2.50 (1.60, 3.60)	Z=-0.35	0.729
NK (%), M (Q₁, Q₃)	9.38 (6.00, 15.21)	10.76 (7.05, 16.84)	7.95 (4.87, 12.59)	Z=-6.17	< 0.001
B cell (%), M (Q₁, Q₃)	21.25 (15.14, 29.46)	18.09 (13.83, 23.73)	27.95 (19.72, 39.64)	Z=-10.27	< 0.001
SEX, n (%)				χ²=0.00	0.991
Female	266 (38.05)	158 (38.07)	108 (38.03)		
Male	433 (61.95)	257 (61.93)	176 (61.97)		
LOS, M (Q₁, Q₃)	7.00 (5.00, 9.00)	6.00 (5.00, 8.00)	9.00 (7.00, 13.00)	Z=-12.55	< 0.001
Underlying disease, n (%)	258 (36.91)	143 (34.46)	115 (40.49)	χ²=2.64	0.104
Co-infection, n (%)	385 (55.08)	196 (47.23)	189 (66.55)	χ²=25.44	< 0.001
Bacteria, n (%)	174 (24.89)	100 (24.10)	74 (26.06)	χ²=0.35	0.556
Virus, n (%)	121 (17.31)	47 (11.33)	74 (26.06)	χ²=25.56	< 0.001
MP, n (%)	197 (28.18)	103 (24.82)	94 (33.10)	χ²=5.71	0.017
FLU, n (%)	40 (5.72)	16 (3.86)	24 (8.45)	χ²=6.60	0.010
RSV, n (%)	34 (4.86)	16 (3.86)	18 (6.34)	χ²=2.25	0.134
EB, n (%)	9 (1.29)	3 (0.72)	6 (2.11)	χ²=1.59	0.208
Respiratory failure, n (%)	33 (4.72)	1 (0.24)	32 (11.27)	χ²=45.58	< 0.001
Heart failure, n (%)	33 (4.72)	1 (0.24)	32 (11.27)	χ²=45.58	< 0.001
Sepsis, n (%)	40 (5.72)	23 (5.54)	17 (5.99)	χ²=0.06	0.804

t: t-test, Z: Mann-Whitney test, χ²: Chi-square test

SD: standard deviation, M: Median, Q₁: 1st Quartile, Q₃: 3st Quartile

### Ridge regression variable screening and multifactor logistic regression analysis

**Fig. 2:**
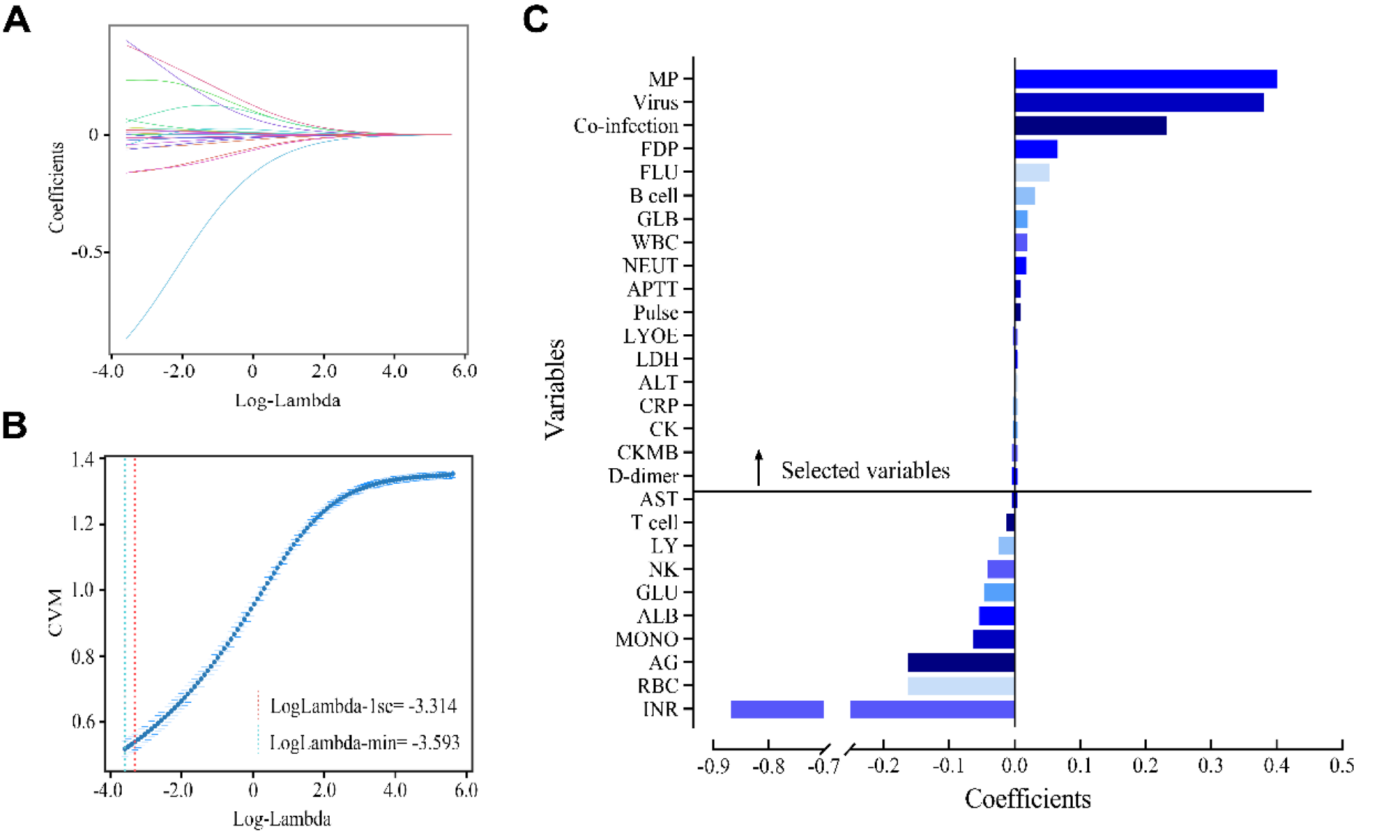
Filtering of variables by Ridge regression to minimize the loss function to determine the optimal model parameters. **a**: Plot of Ridge regression lambda value coefficients; **b**: Plot of Ridge binomial regression lambda value mean square errors; **c**: Correspondence of the Ridge regression minimum lambda value to the coefficient value, and the variable of Coefficient > 0 can be considered

We collected diverse variables in the EHR system, obtained many statistically significant variables through differential analysis, and then performed variable screening using the Ridge regression algorithm. With a minimum cross-validation error, the optimal lambda value for Ridge regression is 0.0275. From this, the variables of Coefficient > 0 are MP, co-infection, co-infection virus, LOS, FDP, B cell, GLB, WBC, NEUT, APTT, Pulse, LDH, ALT, CRP, CK, CKMB and D-dimer (Fig. 2). Then, multivariate logistic regression analysis was performed with whether SAP occurred (yes = 1, no = 0) as the dependent variables, and 13 variables screened by Ridge regression were used as independent variables. The results showed that NEUT, D-dimer, FDP, B cell, and LDH were independent risk factors for SAP in children (Table 3).

**Table 3:** Multivariate logistic analysis of SAP in children

Variables	Beta	S. E	Wald	*P*	aOR (95%CI)
**NEUT**	**0.083**	**0.015**	**29.444**	**< 0.001**	**1.086 (1.054–1.119)**
**LDH**	**0.008**	**0.002**	**25.504**	**< 0.001**	**1.008 (1.005–1.011)**
**D-dimer**	**0.005**	**0.001**	**22.371**	**< 0.001**	**1.005 (1.003–1.007)**
**B cell**	**0.073**	**0.015**	**24.974**	**< 0.001**	**1.076 (1.046–1.107)**
**FDP**	**0.293**	**0.132**	**4.898**	**0.027**	**1.341 (1.034–1.738)**
CK	0.003	0.002	3.415	0.065	1.003 (1.000–1.007)
WBC	0.018	0.013	1.933	0.164	1.018 (0.993–1.044)
Pulse	0.009	0.009	0.912	0.339	1.009 (0.991–1.028)
APTT	0.017	0.027	0.367	0.545	1.017 (0.963–1.073)
Virus (0 = no, 1 = yes)	0.605	0.665	0.829	0.363	1.831 (0.498–6.737)
MP (0 = no, 1 = yes)	0.565	0.541	1.092	0.296	1.760 (0.610–5.079)
LYOE	0.085	0.100	0.723	0.395	1.089 (0.895–1.326)
Co-infection (0 = no, 1 = yes)	0.392	0.549	0.510	0.475	1.480 (0.505–4.343)
ALT	0.002	0.005	0.203	0.653	1.002 (0.993–1.012)
CKMB	-0.007	0.010	0.425	0.514	0.993 (0.973–1.014)
GLB	-0.008	0.031	0.060	0.807	0.992 (0.933–1.055)
CRP	0.002	0.007	0.081	0.776	1.002 (0.989–1.015)
FLU (0 = no, 1 = yes)	-0.183	0.761	0.058	0.810	0.833 (0.187–3.700)

### Construction and effectiveness evaluation of a predictive model for SAP in children

**Fig. 3:**
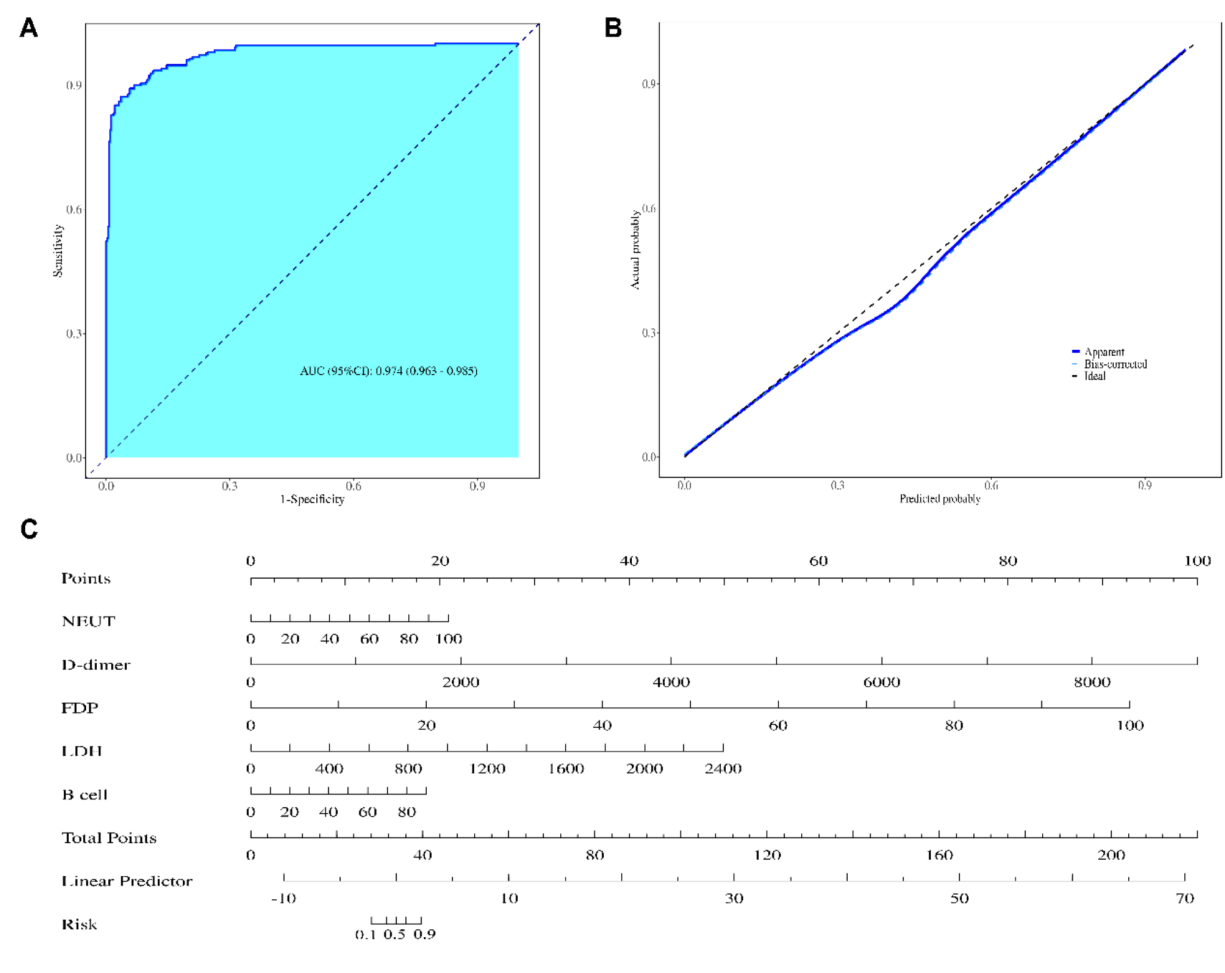
Construction and verification of the nomogram. **a**: ROC curve of the nomogram. The model showed good discriminative ability, with an AUC of 0.974 (95% CI: 0.963–0.985). **b**: Calibration curve of the nomogram. The value of the Hosmer-Lemeshow test was 0.547, indicating that the deviation between the predicted and actual values of the model is slight and that the model has good predictive value. **c**: The nomogram. The total score for individual patients with AVP was calculated by addition of the scores corresponding to each predictive point; the higher the total score, the higher the risk of progression to SAP

According to the analysis results, a nomogram model was constructed, the ROC curve was plotted using the constant term and regression coefficient, the AUC of the curve was 0.974 (95% CI: 0.963–0.985), and the P value of the Hosmer-Lemeshow test was 0.547 (*P* > 0.05), indicating that the model had a good calibration degree and strong prediction ability (Fig. 3).

## Discussion

Infection with HAdV is one of the leading causes of underlying pneumonia in children and is often presented with symptoms such as high fever. The disease progresses rapidly and frequently; one in three patients experiences severe disease [11]. Adenovirus infection is reported to cause various diseases with varying severity in immunocompromised people, depending on the degree of immunosuppression. Children have incompletely developed immune systems and are more likely to acquire severe infection with HAdV types 3 and 7, which often cause SAP [5, 12].

In afflicted children, SAP can cause dysfunctions of many systems, including the digestive, circulatory, and nervous systems, thereby severely burdening the family and society. Therefore, it is crucial to diagnose AVP in children early, intervene as early as possible, and provide appropriate treatment to prevent the occurrence of SAP [13]. In the present study, the disease was more common in males than in females (male-to-female ratio was 1.63:1), and the average age of the patients was 35 months. These observations align with those previously reported, where HAdV infection was more frequent among children under four years of age, probably owing to the incomplete development of their immune functions [14].

HAdV infection is often accompanied by co-infections with other pathogens, such as bacteria and mycoplasma, which might lead to severe clinical symptoms and complications in hospitalized patients and is usually associated with a poor prognosis. In this study, 55.08% (385/699) of patients reported co-infections. The co-infection rate in the SAP group alone was 66.55% (189/284), which is higher than that reported by Esposito et al. (49.2%) [15]. Herein, *MP* (51.16%) was the main infectious agent detected in 385 children with mixed infections. Still, many of these patients were infected with two or more pathogens. In recent years, pneumonia caused by HAdV combined with mycoplasma infection has attracted scientific attention, given the severe clinical symptoms and complications associated with it and the challenges faced during clinical diagnosis and prognosis [16]. Therefore, in routine clinical practice, pediatricians should test for infections with mycoplasma and other pathogens and promptly commence symptomatic treatment against co-infected pathogens.

The research used Ridge filtering and logistic analysis of basic clinical and laboratory indicators in children with AVP. Five variables, namely, NEUT, D-dimer, FDP, B cells, and LDH, were identified as risk factors for the development of SAP. LDH is a critical metabolic enzyme in human cells that is generally highly active in tissues and very sensitive to tissue damage. In this study, the level of LDH was significantly higher in the SAP group than in the AVP group. Children with SAP are reported to experience respiratory failure, an intense inflammatory response, and microcirculatory dysfunction. Stimulating inflammatory factors and oxygen-free radicals can potentially induce macrophages’ apoptosis within the pulmonary alveoli, eventually causing damage to cells and the lung tissue [17]. Respiratory failure can lead to myocardial hypoxia, secretion of myocardial enzymes, and damage of the lung tissue, and might cause different degrees of damage to cardiomyocytes. The level of LDH within the blood would sharply rise in response to the damage caused to cardiomyocytes. Reports have highlighted high serum LDH levels as one of the biomarkers determining the prognosis of severe infection. As the severity of pneumonia is closely related to impairment in cardiac function, doctors must monitor the changes in cardiac function indicators in children during the disease. This observation might suggest early identification of SAP and evaluation of prognosis.

As AVP progresses to SAP, the body secretes several inflammatory factors that may cause disorders in the blood coagulation and fibrinolytic systems. D-dimer is a fibrin degradation product and, together with FDP, is considered a key indicator of coagulation dysfunction, such as hypercoagulability and hyperfibrinolysis. The level of D-dimer increases in response to the occurrence of coagulation disorders in the body. Herein, the levels of D-dimer and FDP significantly increased in the SAP group. Studies have found that severe viral pneumonia stimulates fibrin lysis [18]. D-dimer is a direct product of fibrin cleavage and exhibits better diagnostic specificity. Given its specificity, the D-dimer level is thought to elevate in the early stages of viral infections and then increase exponentially in severe infections, consistent with that observed in the present study [19]. Coagulation function depicts a close correlation with the severity of pneumonia, and its timely testing when children with illness get hospitalized can allow monitoring of disease severity. Meanwhile, severely ill children can be treated with a combination therapy with different anticoagulants to positively prevent the formation of microthrombus and lower the probability of serious complications [20, 21].

Compared with other infections, HAdV infection is known to cause a severe inflammatory response in the lungs and throughout the body. Children’s immune function is incompletely developed, so their body exerts a more extended immune response after infection than adults [22]. Excessive inflammatory response predisposes children with SAP to systemic inflammatory response syndrome (SIRS), which may progress to circularity disorder and multiple systemic organ failure (MSOF). The acute clinical picture of AVP infection becomes more evident as the disease progresses, consistent with an increase in the levels of WBC and NEUT [23]. WBC is a critical player in the body’s defense system but is also susceptible to high body temperature, mental state, trauma, and other aspects. Hence, the detection specificity of WBC count is not high, and doctors may need to make a comprehensive evaluation while diagnosing the disease [24]. Neutrophil extracellular traps (NETs) are essential in the early stages of adenovirus infection. Upon adenovirus invasion, pattern recognition receptors (PRRs) activate neutrophils to recognize the virus and produce NETs, an innate immune response that prevents the virus from spreading in the body at the start of the infection [25, 26]. However, the overactivation and release of neutrophils and NETs observed with an increase in the severity of the disease may exacerbate the inflammatory response and damage the lungs, even causing hypoxemia and ARDS [27, 28]. Therefore, one might closely relate NEUT level to the occurrence of SAP in children. Evidence suggests that the pathogenic mechanism underlying SAP in children involves HAdV-mediated changes in their immune functions. Adenovirus infection can affect the functioning of the immune system and histiocytes and cause damage to multiple organs, eventually leading to a sharp decrease in the production of lymphocytes [29]. We found abnormal levels of lymphocytes in children with SAP, which is reflective of the changes in their immune system and indicative of immune disturbances. The decrease in the proportion of T cells and NK cells is attributed to the enhancement in apoptosis signaling in immune cells; however, the significant increase in the proportion of B lymphocytes may be related to their direct activation after HAdV infection [30, 31].

In this study, the area under the ROC curve was 0.974 (95% CI, 0.963–0.985), and the Hosmer-Lemeshow test P was 0.547 (*P* > 0.05), indicating that the information on children was fully extracted and that the actual number of occurrences was consistent with the predicted number of occurrences. Thus, the model showed good discrimination and accuracy and had good clinical application value. For high-risk children with SAP, clinicians need to dynamically monitor the changes in the disease state, assess the severity of the patient’s condition, perform early intervention, and provide symptomatic treatment to reduce the mortality rate and sequelae.

The present study has some limitations. First, due to laboratory limitations, we only diagnosed children with adenovirus infection and did not distinguish between specific serotypes. Hence, we could not provide a detailed observation of variations in clinical features and factors influencing SAP based on serotypes. Second, this was a single-center retrospective study of children in Nanjing, China, and the model was developed using a retrospective study, so the results may be biased. Therefore, there is a need for prospective and multicenter studies to validate and improve the model.

## Conclusions

A predictive model for children with SAP was constructed using a retrospective investigation of hospitalized children, resulting in the identification of NEUT, D-dimer, FDP, the B-cell ratio, and LDH levels as risk factors. Although the model requires validation in a prospective study, the initial evaluation is that the model allows for a rapid risk assessment of SAP based on all signs on admission and basic laboratory test indicators for the timely identification of high-risk children.

## Data Availability

The datasets generated and analysed during the current study are not publicly available due patient data comes from the hospital’s electronic medical record system but are available from the corresponding author on reasonable request.
